# Self-management strategies amongst Australian women with endometriosis: a national online survey

**DOI:** 10.1186/s12906-019-2431-x

**Published:** 2019-01-15

**Authors:** Mike Armour, Justin Sinclair, K. Jane Chalmers, Caroline A. Smith

**Affiliations:** 1 NICM Health Research Institute, Western Sydney University, Building 5, Campbelltown Campus, Locked Bag 1797, Penrith, Sydney, NSW 2751 Australia; 2 School of Science and Health, Western Sydney University, Sydney, Australia

**Keywords:** Self-management, Self-care, Endometriosis, Exercise, Heat, Cannabis

## Abstract

**Background:**

Endometriosis has a significant negative impact on the lives of women, and current medical treatments often do not give sufficient pain relief or have intolerable side effects for many women. The majority of women with primary dysmenorrhea use self-management strategies (including self-care techniques or lifestyle choices) to help manage period related symptoms, but little is known about self-management in women with endometriosis. The aim of this survey was to determine the prevalence of use, safety, and self-rated effectiveness of common forms of self-management.

**Methods:**

A cross-sectional online survey was distributed via social media using endometriosis support and advocacy groups in Australia between October and December 2017. Women were eligible to answer the survey if they were 18–45, lived in Australia, and had a confirmed diagnosis of endometriosis. Survey questions covered the types of self-management used, improvements in symptoms or reduction in medication, and safety.

**Results:**

Four hundred and eighty-four valid responses were received. Self-management strategies, consisting of self-care or lifestyle choices, were very common (76%) amongst women with endometriosis. The most common forms used were heat (70%), rest (68%), and meditation or breathing exercises (47%). Cannabis, heat, hemp/CBD oil, and dietary changes were the most highly rated in terms of self-reported effectiveness in pain reduction (with mean effectiveness of 7.6, 6.52, 6.33, and 6.39, respectively, on a 10-point scale). Physical interventions such as yoga/Pilates, stretching, and exercise were rated as being less effective. Adverse events were common, especially with using alcohol (53.8%) and exercise (34.2%).

**Conclusions:**

Self-management was very commonly used by women with endometriosis and form an important part of self-management. Women using cannabis reported the highest self-rated effectiveness. Women with endometriosis have unique needs compared to women with primary dysmenorrhea, and therefore any self-management strategies, especially those that are physical in nature, need to be considered in light of the potential for ‘flare ups’.

**Electronic supplementary material:**

The online version of this article (10.1186/s12906-019-2431-x) contains supplementary material, which is available to authorized users.

## Background

Chronic pelvic pain is pain in the pelvis of greater than 6 months duration that is severe enough to cause functional disability or require medical intervention [1]. Worldwide prevalence rates range between 5.7 and 26.6% [2]. Endometriosis is the presence of endometrial tissue outside the uterine cavity and is the most common cause of chronic pelvic pain [3] with 24 to 40% of women with chronic pelvic pain diagnosed with endometriosis [4, 5]. A recent cohort study of Australian women aged 34–39 years had a prevalence of confirmed endometriosis of 3.7% [6]. Endometriosis related chronic pelvic pain includes a variety of pain symptoms including dysmenorrhea (period pain), dyspareunia (pain during sexual intercourse), dyschezia (pain on bowel motions), and dysuria (pain on urination). In addition to severe pelvic pain [4], endometriosis reduces quality of life and increases absenteeism at work or school [7]. Endometriosis impacts women’s health and wellbeing, including social activities [7], mental and emotional health [8], work and finances [7], and sexual relationships [9], and has been shown to reduce physical quality of life similar to that of cancer patients [7].

Current non-surgical treatments such as non-steroidal anti-inflammatories, oral contraceptive pills, and hormonal treatments have limited effectiveness [10] and the side effect profile is bothersome, with discontinuation rates of between 25 and 50% [11]. Because of this, it is likely that women will use self-care or lifestyle interventions as part of their self-management strategies, to manage either some of their symptoms and/or some of the side effects from the medications, either over the counter or prescribed, used to manage their endometriosis. Use of self-management strategies are incredibly common in women with dysmenorrhea [12, 13]. Women in Australia with symptoms of endometriosis do use complementary therapies [6], and there is evidence of effectiveness of several of these self-management therapies or lifestyle interventions in managing endometriosis symptoms, including dietary changes [14] and yoga [15]. Evidence from other ongoing participant centric research such as ‘Citizen Endo’ [16] suggests that women are using other methods such as cannabis and alcohol to help manage their pain. There is preliminary evidence that the endocannabinoid system can play an important role in managing endometriosis pain [17], and therefore women may be self-medicating with cannabis products to reduce dependence on opioid based pain relief. If women in the community are using self-management and finding it effective this will help direct future research efforts into both studying effectiveness and increasing awareness about effective self-management.

The aim of this survey was to determine the prevalence of use, safety, and self-rated effectiveness of common forms of self-management in women with endometriosis.

## Methods

An online questionnaire was developed by the research team in conjunction with 19 women with endometriosis and hosted on the Qualtrics platform (Qualtrics Ltd). Self-management was defined as physical or psychological techniques that women could administer or perform themselves or lifestyle interventions (such as dietary changes, alcohol or cannabis usage) that were undertaken specifically for the management of endometriosis symptoms. An initial list was compiled from endometriosis support online discussions forums and those that had been mentioned as part of the Citizen Endo project [16]. This list was then presented and discussed during two 90-min focus groups that were run in Sydney, Australia in July 2017. Focus groups included 19 women aged 21–45 with Endometriosis. The self-management strategies that women in the focus group had used themselves or that they thought were commonly used in the endometriosis community were included in the questionnaire. Broad categories (e.g. exercise) were adopted in the questionnaire due to the large number of self-management interventions reported by women and to reduce participant burden. All measures were self-reported and required recall over the past 6 months. The questionnaire collected demographics, use of self-management techniques in the previous 6 months, reasons for non-usage of self-management, type and frequency of self-management used, adverse events, self-rated effectiveness and any reduction in endometriosis related medication usage. The Pelvic Pain Impact Questionnaire (PPIQ) was included to assess the severity of pelvic pain in the sample [18]. Five young Australian women (aged 20–27) piloted the survey prior to publication, and minor amendments to wording to improve clarity were made.

The survey took approximately 15–20 min to complete. Features were enabled within Qualtrics that prevented multiple completions from either a single IP address or the same computer. A full copy of the survey can be found in Additional file 1. This article provides an overall summary and comparison of all the surveyed self-management strategies. In depth analysis, including costing, on the highest rated forms of self-management will be published separately.

Women were eligible to participate in the survey if they were aged 18–45, currently living in Australia, and had a diagnosis of endometriosis, confirmed by a laparoscopy within the last 5 years.

Recruitment was conducted via a direct link to the survey and an invitation to participate distributed via the social media platforms (Facebook, Twitter, and Instagram) of Endometriosis Australia and EndoActive, the two Endometriosis advocacy and advice groups in Australia with the most followers on social media. The total combined reach of these patient advocacy organisations on social media is just over 33,000 followers. Each organisation made two social media posts regarding the survey, the first post in October 2017, and the second post 3–5 weeks after the first. The survey link was active from October 2017 to December 2017, for a total of 6 weeks. Data collection was closed once there had been no new responses for 10 days. Data was analysed using SPSS v24 (IBM Corporation). Descriptive statistics were presented as means and standard deviations for continuous data or number and percentages for categorical data. Inferential statistics for between group comparisons were performed using a one-way ANOVA. Statistical significance was set at *p* < 0.05. Missing data was not replaced. Free text responses on the details of adverse events were imported and then categorized using an Excel spreadsheet (Excel 2016, Microsoft Corporation). Adverse events were broadly categorized based on the free text responses; comments using language such as tired, or exhausted were categorized as fatigue, while sleepiness or hard to stay awake were categorized as drowsiness. Where women used the term ‘flare-up’ it was kept as its own category as this language is often used to describe an increase in not only pain but all endometriosis symptoms. Where ‘flare-up’ was not used, but pain increases were described, responses were categorized based on where the pain was located (if mentioned). Adverse event categories were classed as ‘common’ if more than 25% of women reported them.

This survey was approved by the Western Sydney University Human Research Ethics Committee, approval number H12394, approved 23rd October 2017.

## Results

Five hundred and ninety women completed the survey. Ninety-six of the responses were incomplete (less than 25% of the survey complete) and ten responses were excluded as they either did not live in Australia or were outside the age range. A total of 484 responses were suitable for inclusion in the analysis and were used as the denominator for analysis. Table 1 outlines the demographic characteristics of the participants.

**Table 1:** Characteristics of survey respondents (*N* = 484)

Age (y)	Mean (SD)
	31 (7.4)
PPIQ Scores (0–4) (listed greatest to lowest impact)	N (%)
Stomach/GI function	2.6 (0.5)
Energy levels	2.6 (0.4)
Mood	2.4 (0.4)
Clothing	2.3 (0.4)
Physical activity	2.3 (0.3)
Work/School	2.2 (0.3)
Sleep	2.1 (0.3)
Sitting	1.5 (0.2)
Total	17.9 (2.7)
Region	N (%)
Urban	374 (78%)
Rural	103 (21%)
Remote	3 (< 1%)
Used self-management in last 6 months?	N (%)
	371 (76%)
Self-management used in last 6 months (listed most to least common)	N (%)
Heat	259 (70%)
Rest	252 (68%)
Meditation/Breathing	175 (47%)
Dietary choices (such as gluten free, vegan)	163 (44%)
Exercise	158 (42%)
Stretching	148 (40%)
Yoga/Pilates	131 (35%)
Massage	118 (32%)
Herbal medicines	61 (16%)
Alcohol	51 (14%)
Cannabis	48 (13%)
Acupressure	29 (8%)
Cold	18 (5%)
Hemp oil/CBD oil	12 (3%)
Taichi/Qigong	8 (2%)

### Use of self-management

The most commonly used forms of self-management were heat (70%), rest (68%), and meditation or breathing exercises (47%). For those women who did not use self-management (Table 2) the most common reasons for their non-use was that they did not have enough information to make a decision (36%) and the time commitment (28%) or cost (28%) involved.

**Table 2:** Reasons for non-use of self-management (*N* = 109), listed most to least commonly reported

Reason	N (%) ^a^
Not enough information to make decision	39 (36%)
Time commitment	31 (28%)
Cost	30 (28%)
Ineffective in previous experience	29 (27%)
Difficulty accessing	19 (17%)
Other	15 (14%)

^a^More than one response was allowed, therefore percentages sum to greater than 100

### Effectiveness of self-management

Women’s self-reported effectiveness (based on a 0–10 score, with 0 being ineffective and 10 being extremely effective) for each self-management intervention (see Table 3) showed that cannabis (7.6 ± 2.0), heat (6.5 ± 1.7), dietary choices (6.4 ± 2.4), hemp/CBD oil (6.3 ± 3.0) and acupressure (6.3 ± 1.6) were the most effective. Physical interventions such as yoga/Pilates (4.5 ± 2.0), stretching (4.6 ± 2.1) and exercise (4.9 ± 2.4) were rated as being less effective. A comparison of the different types of diet (e.g. paleo, vegan, FODMAP) did not show any relationship between a specific diet and self-reported improvement (*p* = 0.097).

**Table 3:** Level of self-reported pain relief from self-management modalities, listed from greatest to smallest reported pain reduction

Modality used for self-management	Pain relief (0–10 scale) Mean (SD)
Cannabis	7.6 (2.0)
Heat	6.5 (1.7)
Dietary choices (such as gluten free, vegan)	6.4 (2.4)
Hemp oil/CBD oil	6.3 (3.0)
Acupressure	6.3 (1.6)
Cold	5.5 (2.7)
Massage	5.5 (2.1)
Rest	5.3 (2.1)
Exercise	4.9 (2.4)
Herbal medicines	4.8 (2.5)
Alcohol	4.7 (2.3)
Stretching	4.6 (2.1)
Meditation/Breathing	4.6 (2.1)
Yoga/Pilates	4.5 (2.0)
Taichi/Qigong	4.0 (1.7)

When women were asked about the effect self-management had on their need for medications needed to manage their endometriosis symptoms, the most effective was cannabis. Fifty six percent of cannabis users reported being able to reduce their endometriosis related medication by more than 50% and another 27% percent of users reported being able to reduce medication by 25–50%. Other self-management practices were considerably less effective in medication reduction; a third of CBD or hemp oil users reported being able to reduce their endometriosis related medication by 50% or more, while only 18% of those who used a specific diet reported being able to reduce their endometriosis related medication by 50% or more. The full list of each self-management option and its effect on medication is reported in Additional file 2: Table S1.

### Adverse events during self-management

Adverse events varied considerably between self-management interventions (Table 4). Alcohol usage showed the greatest number of self-reported adverse events, with just over half (52.8%) of users reporting an adverse reaction. The most common reported events were viesalgia (hangovers) and increases in pain and fatigue after alcohol usage. Exercise also showed a large number of adverse events, with just over one third of women reporting adverse events (34.2%). The most common adverse events were increased pelvic pain (especially cramping pain), increased frequency of ‘flare ups’, and increased fatigue. Heat users reported adverse events (15.9%), most commonly burns. Increased pelvic pain was reported in 15.9% of yoga and Pilates, but these reports were mostly linked to Pilates rather than yoga.

**Table 4:** Adverse events from self-management modalities, listed from most to least commonly reported

Modality used for self-management	Adverse event rate % (of women using that modality)	Most common reported types of AE^a^
Alcohol	52.8	Hangover symptoms, increased pain, increased fatigue
Exercise	34.2	Increased adhesion/pelvic pain, increased fatigue, increased flare ups
Yoga/Pilates	15.9	Increased adhesion/pelvic pain
Heat	15.9	Burns (including blistering)
Stretching	14.8	Increased adhesion/pelvic pain, increased nausea, increased flare ups
Cannabis	10.2	Drowsiness, Increased anxiety, tachycardia
Hemp oil/CBD oil	8.3	N/A
Rest	7.3	Increased fatigue, increased depression
Acupressure	7.1	N/A
Massage	6.8	N/A
Dietary choices (such as gluten free, vegan)	5.9	Gastrointestinal upset
Cold	5.6	N/A
Meditation/Breathing	3.4	N/A
Herbal medicines	3.2	N/A
Taichi/Qigong	0	N/A

^a^Categorized from the free text responses provided. Responses were considered ‘common’ when 25% or more of the respondents included them. N/A denotes where (due to the small number of responses) there was no AE that met the 25% threshold

## Discussion

The use of self-management strategies, especially non-pharmacological practices, in chronic illnesses is common [19]. Women with endometriosis often feel frustrated at the lack of effective medical treatments and therefore turn to self-management [20] as one of the coping strategies to manage their condition [21]. Women with endometriosis often feel disempowered [20], and self-management strategies may help them feel a sense of agency and empowerment [22]. Our survey findings support this: both self-management techniques and lifestyle interventions or modifications are a common and important part of Australian women’s self-management strategy when dealing with the symptoms of endometriosis.

Usage of self-management was high in women with endometriosis, with overall rates of self-management being similar to those observed in women with primary dysmenorrhea [13]. The use of self-management techniques that includes components that may be considered to be ‘complementary’ or ‘alternative’ such as yoga or acupuncture can form an important part of self-management for women, and are often [23], but not always [20], seen as an adjunct rather than a replacement for, mainstream medical care.

The mean age of women in our study (31 years old) is similar to other studies on women with endometriosis in Europe (33 years) [7], South Africa (33 years) [21] and previous research undertaken in Australia (31 years) [24]. The impact of endometriosis on the lives of women in this study is comparable to worldwide impact reports [18]. The greatest impacts were in energy levels, gastrointestinal function, and mood, which may directly relate to the choices of self-management strategies that women make. For example, women with endometriosis may use yoga, cannabis or hemp/CBD oil to improve their mood and make dietary changes to self-manage gastrointestinal problems.

Diet, while not as commonly used as rest and heat, was used by almost half the women in the survey. Diet had high self-reported improvement scores and examination of the types of diets used showed there was significant diversity; with paleo, vegan, gluten free, FODMAP and Mediterranean diets being the most common sub-types of diet. Given the significant proportion of women with endometriosis who also have gastrointestinal [25] and IBS-like symptoms [14], dietary changes, such as a FODMAP diet, may reduce pelvic pain symptoms that could be exacerbated by IBS or gastrointestinal symptoms. This is likely to occur via a reduction in intestinal distention and subsequent reduction in visceral nerve activation [14]. In our survey, there was no specific sub-type of diet reported that was related to a significantly greater self-rated improvement. This appears to be in line with current reviews which find there are no consistent dietary predictors for endometriosis [26].

Heat was the only modality that was both commonly used and rated as effective by women. There are no studies looking specifically at heat for endometriosis related pain but previous research provides evidence that heat reduces primary dysmenorrhea [27]. Heat may work via both increasing blood flow in the abdominal area [28] and by the ‘gate control’ theory of pain inhibition, where topical heat activates thermoreceptors, inhibiting concurrent nociceptive signals reaching the brain [27]. However, despite its effectiveness, a significant number of women reported adverse events with heat, most commonly burns. Therefore, consideration should be given to the use of heat patches that deliver controlled heat at a safe temperature.

The first reported use of cannabis being used as a medicine for female reproductive complaints was in China ca. 2700 BCE. More recently, phytochemical constituents within the plant such as the cannabinoids ∆^9^-Tetrahydrocannabinol (THC) [29, 30], ∆^9^-Tetrahydrocannabivarin (THCV) [31] and Cannabidiol (CBD) [32] have demonstrated noted pharmacological activity, specifically analgesic and anti-inflammatory effects. Cannabis, while only used by 13% of women in this survey, had the highest pain relief score and greatest reduction in medication usage of any strategy assessed. This latter finding is mirrored in other international cannabis studies showing a reduction in pharmaceutical medication usage for pain management, with recent evidence demonstrating that cannabis may assist in de-prescribing from pharmaceutical medication, particularly opiates and benzodiazepines, in what is dubbed the substitution effect [33]. Whilst further studies specific to the endometriosis population are required, considering the known abuse, risk of addiction and overdose mortality rates with opiate medications [34], coupled with recent evidence suggesting medicinal cannabis can reduce prescription opiate overdose mortality rates significantly [35], quality assured medicinal cannabis may play a role as both an adjunct analgesic and harm reduction agent. Australia introduced the Narcotic Drugs Regulation in December 2016 to legalise cannabis for medicinal use, with medical practitioners being able to prescribe cannabis products through various avenues including the Special Access Scheme and Authorised Prescriber pathways. Whilst government pathways do not preclude endometriosis or pelvic pain patients from access in Australia, current numbers of approved patients based on clinical indication is suggestive that survey respondents were utilising illicit cannabis.

Both rest and physical activity have been reported as effective self-management practices in women with primary dysmenorrhea [12, 36]; however, the findings in the current survey did not find these methods effective for women with endometriosis, and even found high levels of adverse events due to physical activity. That rest and physical activity are not effective self-management methods in endometriosis is not altogether unsurprising; rest is considered a passive treatment that is ineffective in many chronic pain conditions [37, 38], as it is thought to promote illness behaviour [39] and fear-avoidance behaviour [40]. Similarly, engaging in vigorous physical activity after a period of rest or reduced levels of activity is ineffective in treating chronic pain conditions [41] and can often make pain symptoms worse – inducing a so called ‘flare up’. In women with endometriosis, vigorous exercise may exacerbate pelvic muscle spasms that are commonly seen in these women [42]. While physical activity is essential for chronic pain recovery [43], it needs to be gradual (graded exposure) to avoid flare ups, to improve physical activity tolerance, and to allow individuals to return to their usual level of daily activity [41].

There are clear strengths to this study. First, women must have had endometriosis diagnosed via laparoscopy within the previous 5 years, providing a specific population. Despite laparoscopic investigation being the gold standard in diagnosing endometriosis, some research studies continue to include women with diagnoses of endometriosis based on symptomology reports alone, leading to potential bias from differential or overlapping conditions. Second, the sample size obtained was large in comparison to other survey methodologies with similar populations. Finally, the online survey methodology allowed participants to have anonymity. Greater anonymity improves the willingness of participants to disclose sensitive information [44], such as drug use, which increases the confidence that the present results truly reflect all self-management measures taken by women with endometriosis.

Importantly, the findings from this study should be taken into consideration with the study limitations. Due to the use of social media as a recruitment tool, calculating a response rate is not possible, therefore any generalisability of these findings must be done with caution. Women recruited via support/advocacy groups often have more severe symptoms than those recruited via other methods [45]; however, our sample had very similar PPIQ scores to a large international sample [18]. A small portion of the population came from remote regions of Australia. Arguably, women in remote regions may use different self-management measures due to the decreased availability of medical resources; however, the small sample size obtained did not allow for any analyses that might detect differences. In addition, the questionnaire offered no free response section in which women could nominate other self-management measures not listed. There is the potential that women with endometriosis use additional or alternative self-management measures, but this information was not able to be captured by this questionnaire. Reporting of reduction in endometriosis related medication did not collect data on what class of medication (e.g. analgesics) were being reduced. To reduce survey length, broad categories were used and therefore comparisons between specific sub-types (e.g. walking vs vigorous exercise) was not possible. Combination of yoga and Pilates into one category may obscure the effect of these two practices, as demonstrated by the free text reporting for adverse events with Pilates. Finally, all measures were self-reported, therefore all indications of effectiveness and adverse events are based on women’s own recall, and this may lead to either over or underestimation of benefits and harms.

## Conclusions

Given the lack of a ‘cure’ for endometriosis, effective self-management techniques and lifestyle changes may play an important role in ongoing self-management by empowering women to take more control over their own health and providing an effective adjunct to their current treatment regimes. Women with endometriosis have unique needs compared to women with primary dysmenorrhea, and therefore any self-management practices, especially those that are physical in nature, need to be considered in light of the potential for ‘flare ups’. Cannabis users report significant effectiveness for reducing endometriosis related pain and related symptoms, however the number of women using it is small and outcomes all self-reported, therefore future clinical trials in this area are required to determine any possible role in endometriosis management utilising legally obtained and quality assured medicinal cannabis. Medicinal cannabis is becoming available in a growing number of locations but remains illegal for treating pelvic pain in many countries. Therefore, cannabis should only be considered as a possible self-management option by those who can obtain medicinal cannabis through legal means.

## Additional files

Additional file 1: A Survey tool. Full copy of survey used for data collection. (PDF 284 kb)

Additional file 2: **Table S1.** Reduction in medication usage due to the use of self-management. Changes in endometriosis related medication for all self-management modalities. (DOCX 22 kb)

## Abbreviations

CBD: Cannabidiol
FODMAP: Fermentable oligosaccharides, disaccharides, monosaccharides and polyols
PPIQ: Pelvic pain impact questionnaire
THC: Tetrahydrocannabinol
