# Supplementary material for: Self-management strategies amongst Australian women with endometriosis: a national online survey
Source: BMC Complement Altern Med. 2019 Jan 15;19:17. doi: 10.1186/s12906-019-2431-x (PMC6332532; doi:10.1186/s12906-019-2431-x)
Supplement: Supplementary file 1 — A Survey tool. Full copy of survey used for data collection. (PDF 284 kb) [file 12906_2019_2431_MOESM1_ESM.pdf]

Default Question Block

Welcome to the Endometriosis Self-Care survey

What this survey is about

Researchers from Western Sydney University are interested in finding out about what types of non-pharmacological self-care that women with endometriosis use to help manage their endometriosis symptoms. We are also interested to hear from women who are taking medication to treat their endometriosis about any techniques they use to manage side effects of these medications. Non-pharmacological self-care refers to techniques or treatments to help reduce pain or manage other symptoms that you wouldn't normally need a prescription or go to a pharmacist for, and that you would generally either do yourself or could have a friend or family member help you with. Common examples of this would be exercise, yoga postures, breathing or meditation, changing your diet or using heat packs. Less common techniques might be using alcohol or cannabis.

Researchers involved

Dr Mike Armour, Mr Justin Sinclair and Professor Caroline Smith from NICM and Ms Jane Chalmers from the department of Physiotherapy, all from Western Sydney University.
This project has been approved by the Western Sydney University Ethics Committee (HXXXXX).

Eligibility Criteria

To participate in this survey you need to:
- Be currently living in Australia
- Be aged over 18
- Have a diagnosis of endometriosis from your doctor. This is usually done after a laparoscopy.

Content of the survey

The survey will ask about your background and your use (or lack of use) of self-care practices in the management of your endometriosis. Self-care can include breathing exercises, meditation, exercise, yoga, herbal therapies, cannabis or alcohol usage. The survey will ask you questions about your perception of the effectiveness of these self-care techniques, any negative effects from using these and how much money you have spent on these.

What participation involves:

Participation in the study will involve completing an online survey that will take 15-30 minutes. However, this time will vary depending on how many self-care techniques you have used. You will be required to complete the survey in one sitting as we do not collect your IP or use cookies, to protect your privacy

Anonymity

The survey is anonymous and no identifying data such as name, date of birth, address, or email will be requested. We will remove any references to personal information in answers to open-ended questions that might allow someone to guess your identity.

What will happen with the results

It is intended that the results of this study will be published in journal articles, reports, and presented at conferences and other scientific meetings. Reports regarding safety and health effects, using aggregate data, may also be prepared for specific groups/organisations to help facilitate safe practice. Raw data collected will be accessible only by members of the research team.

Voluntary participation

Your participation in this study is completely voluntary. Should you wish to withdraw at any stage before you submit your results, you are free to do so. Unfortunately, once you have submitted your results we are unable to remove them as they are anonymous and so we cannot identify which answers are yours.

How to participate

If you would like to participate, please indicate that you have read and understood this information by clicking the red arrows below. It is important that you complete the survey only once.

Further information

Should you require any further information, or have any concerns, please do not hesitate to contact Dr Mike Armour +61 415363201. If you have any complaints or reservations about the ethical conduct of this research, you may contact the Ethics Committee through Research Engagement, Development and Innovation (REDI) on Tel +61 2 4736 0229 or email humanethics@westernsydney.edu.au. Any issues you raise will be treated in confidence and investigated fully, and you will be informed of the outcome.

By clicking **the red arrow >>** below, you acknowledge that:

- You have read and understand the information above
- Your participation will involve completing an online survey and that the researchers may use the results as described above
- Participation is voluntary
- The survey is anonymous, and any identifying information provided will be edited to ensure confidentiality

Age:

State or territory:

Do you consider yourself to live in:

An urban area
A rural area
A remote or isolated area

In the past month, how much has your pelvic pain affected your:

	None at all	A little	A moderate amount	A lot	A great deal
energy levels?	◯	◯	◯	◯	◯
mood?	◯	◯	◯	◯	◯
sleep?	◯	◯	◯	◯	◯
stomach and intestinal functions?	◯	◯	◯	◯	◯
ability to sit for longer than 20 mins?	◯	◯	◯	◯	◯
ability to perform and function normally at home/school/university/work?	◯	◯	◯	◯	◯
ability to take part in physical activity? (e.g. jogging, yoga, bicycling)	◯	◯	◯	◯	◯
ability to wear certain clothes? (e.g. underwear, tight fitting clothes)	◯	◯	◯	◯	◯

If the following questions apply to you, please answer. If not, please leave these blank. These questions will not be added to your summed score.

	None at all	A little	A moderate amount	A lot	A great deal
During your last period, how much did your pelvic pain affect your ability to use tampons?	◯	◯	◯	◯	◯
In the past month, how much has your pelvic pain affected your levels of intimacy or sexual relationships? (e.g. having sex,	◯	◯	◯	◯	◯

masturbating)

In the last **six months** have you used any non-pharmacological self-care activities or therapies (such as yoga, meditation, special diets, hemp oil etc.) to help you manage your endometriosis symptoms or help reduce the side effects from medication you are taking to help deal with endometriosis (e.g. Endone or other analgesic medication)?

Yes

No

Why don't you use self-care therapies? (select all that apply)

Cost

Time commitment

Tried them and they didn't work

Don't have enough information about them

Can't access them easily

Other

Which of the following non-pharmacological/self-care practices have you used in the last six months for the management of your endometriosis symptoms? (select all that apply)

Yoga or Pilates

Tai Chi/Qi Gong

Meditation or breathing exercises

Rest

Stretching exercises

Exercise or physical activity (such as walking, jogging, swimming or going to the gym)

Heat (eg heat packs, hot water bottle, sauna etc)

Cold packs

Acupressure

Massage

Hemp/CBD oil

Cannabis or cannabis products (prescribed or illicit)

Alcohol

Herbal anti-anxiety/relaxant (e.g. Kava, Passionflower, Camomile)

Dietary therapies (such as FODMAP diet,Paleo diet or Plant based diet)

How often did you practice Yoga or Pilates on average?

Daily or multiple times per day

2-6 times a week

Once a week

Less than once a week

On a scale from 0-10 (0 being totally ineffective and 10 being totally effective), how effective was Yoga or Pilates in reducing your endometriosis related pelvic pain?

Totally ineffective									Totally effective	
0	1	2	3	4	5	6	7	8	9	10

Which symptoms of endometriosis, or side effects of medications that you are taking to treat endometriosis, did Yoga or Pilates reduce and how effective was it?

	Significant improvement (> 50% reduction in severity and/or duration)	Moderate improvement (25-50% reduction in severity and/or duration)	Slight improvement (<25% reduction in severity and/or duration)	No change	Felt worse / symptoms flared up	I don' have th sympto
Nausea / Vomiting	○	○	○	○	○	○
Bowel / Stomach	○	○	○	○	○	○

problems						
Fatigue						
Anxiety						
Depression / Low mood						
Sleep						
Pain on intercourse						
Pain on urination						

Did you experience any adverse reactions from Yoga or Pilates?

Yes

No

What were these adverse reactions?

Did your use of Yoga or Pilates reduce your need to use the normal pharmaceutical therapies you would normally use for your endometriosis symptoms (e.g a reduction in the amount of pain killers you need)?

Yes – significant reduction (50% or more)

Yes – moderate reduction (25-50%)

Yes – minimal reduction (<25%)

No

Other

How much per month, on average, did Yoga or Pilates cost you out of your own money? (This could include a one off costs like a beginners class as well as weekly memberships)

Would you recommend Yoga or Pilates to a friend or family member with endometriosis?

Yes

No

Is there anything else you want to tell us about Yoga or Pilates?

How often did you practice Tai Chi/Qi Gong on average?

Daily or multiple times per day

2-6 times a week

Once a week

Less than once a week

On a scale from 0-10 (0 being totally ineffective and 10 being totally effective), how effective was Tai Chi/Qi Gong in reducing your endometriosis related pelvic pain?

Totally ineffective									Totally effective	
0	1	2	3	4	5	6	7	8	9	10

Which symptoms of endometriosis, or side effects of medications that you are

taking to treat endometriosis, did Tai Chi/Qi Gong reduce and how effective was it?

	Significant improvement (> 50% reduction in severity and/or duration)	Moderate improvement (25-50% reduction in severity and/or duration)	Slight improvement (<25% reduction in severity and/or duration)	No change	Felt worse / symptoms flared up	I don' have th sympto
Nausea / Vomiting	◯	◯	◯	◯	◯	◯
Bowel / Stomach problems	◯	◯	◯	◯	◯	◯
Fatigue	◯	◯	◯	◯	◯	◯
Anxiety	◯	◯	◯	◯	◯	◯
Depression / Low mood	◯	◯	◯	◯	◯	◯
Sleep	◯	◯	◯	◯	◯	◯
Pain on intercourse	◯	◯	◯	◯	◯	◯
Pain on urination	◯	◯	◯	◯	◯	◯

Did you experience any adverse reactions from Tai Chi/Qi Gong?

Yes

No

What were these adverse reactions?

Did your use of Tai Chi/Qi Gong reduce your need to use the normal pharmaceutical therapies you would normally use for your endometriosis symptoms (e.g a reduction in the amount of pain killers you need)?

Yes – significant reduction (50% or more)

Yes – moderate reduction (25-50%)

Yes – minimal reduction (<25%)

No

Other

How much per month, on average, did Tai Chi/Qi Gong cost you out of your own money? (This could include a one off costs like a beginners class)

Would you recommend Tai Chi/Qi Gong to a friend or family member with endometriosis?

Yes

No

Is there anything else you want to tell us about Tai Chi/Qi Gong?

How often did you practice Meditation or breathing exercises on average?

Daily or multiple times per day

2-6 times a week

Once a week

Less than once a week

On a scale from 0-10 (0 being totally ineffective and 10 being totally effective), how effective was Meditation or breathing exercises in reducing your endometriosis related pelvic pain?

Totally ineffective										Totally effective
0	1	2	3	4	5	6	7	8	9	10

Which symptoms of endometriosis, or side effects of medications that you are taking to treat endometriosis, did Meditation or breathing exercises reduce and how effective was it?

	Significant improvement (> 50% reduction in severity and/or duration)	Moderate improvement (25-50% reduction in severity and/or duration)	Slight improvement (<25% reduction in severity and/or duration)	No change	Felt worse / symptoms flared up	I don’[illegible] have th[illegible] sympt[illegible]
Nausea / Vomiting	○	○	○	○	○	○
Bowel / Stomach problems	○	○	○	○	○	○
Fatigue	○	○	○	○	○	○
Anxiety	○	○	○	○	○	○
Depression / Low mood	○	○	○	○	○	○
Sleep	○	○	○	○	○	○
Pain on intercourse	○	○	○	○	○	○

Pain on urination	○	○	○	○	○	○

Did you experience any adverse reactions from Meditation or breathing exercises?

Yes

No

What were these adverse reactions?

Did your use of Meditation or breathing exercises reduce your need to use the normal pharmaceutical therapies you would normally use for your endometriosis symptoms (e.g a reduction in the amount of pain killers you need)?

Yes – significant reduction (50% or more)

Yes – moderate reduction (25-50%)

Yes – minimal reduction (<25%)

No

Other

How much per month, on average, did Meditation or breathing exercises cost you out of your own money? (This could include a one off costs like a beginners mediation class)

Would you recommend Meditation or breathing exercises to a friend or family member with endometriosis?

Yes

No

Is there anything else you want to tell us about Meditation or breathing exercises?

How often did you use rest as a treatment for endometriosis on average?

Daily or multiple times per day

2-6 times a week

Once a week

Less than once a week

On a scale from 0-10 (0 being totally ineffective and 10 being totally effective), how effective was rest in reducing your endometriosis related pelvic pain?

Totally ineffective									Totally effective	
0	1	2	3	4	5	6	7	8	9	10

Which symptoms of endometriosis, or side effects of medications that you are taking to treat endometriosis, did rest reduce and how effective was it?

	Significant improvement (> 50% reduction in severity and/or duration)	Moderate improvement (25-50% reduction in severity and/or duration)	Slight improvement (<25% reduction in severity and/or duration)	No change	Felt worse / symptoms flared up	I don' have th sympto
Nausea /	○	○	○	○	○	○

Vomiting						
Bowel / Stomach problems	○	○	○	○	○	○
Fatigue	○	○	○	○	○	○
Anxiety	○	○	○	○	○	○
Depression / Low mood	○	○	○	○	○	○
Sleep	○	○	○	○	○	○
Pain on intercourse	○	○	○	○	○	○
Pain on urination	○	○	○	○	○	○

Did you experience any adverse reactions from rest?

Yes

No

What were these adverse reactions?

Did your use of rest reduce your need to use the normal pharmaceutical therapies you would normally use for your endometriosis symptoms (e.g a reduction in the amount of pain killers you need)?

Yes – significant reduction (50% or more)

Yes – moderate reduction (25-50%)

Yes – minimal reduction (<25%)

No

Other

On average, over a month, how much did resting cost you in terms of missed work or other income? If there was no cost involved enter 0

Would you recommend rest to a friend or family member with endometriosis?

Yes

No

Is there anything else you want to tell us about resting to help treat endometriosis?

How often did you practice stretching exercises on average?

Daily or multiple times per day

2-6 times a week

Once a week

Less than once a week

On a scale from 0-10 (0 being totally ineffective and 10 being totally effective), how effective were stretching exercises in reducing your endometriosis related pelvic pain?

Totally ineffective										Totally effective
0	1	2	3	4	5	6	7	8	9	10

Which symptoms of endometriosis, or side effects of medications that you are

taking to treat endometriosis, did stretching exercises reduce and how effective was it?

	Significant improvement (> 50% reduction in severity and/or duration)	Moderate improvement (25-50% reduction in severity and/or duration)	Slight improvement (<25% reduction in severity and/or duration)	No change	Felt worse / symptoms flared up	I don' have th sympto
Nausea / Vomiting	○	○	○	○	○	○
Bowel / Stomach problems	○	○	○	○	○	○
Fatigue	○	○	○	○	○	○
Anxiety	○	○	○	○	○	○
Depression / Low mood	○	○	○	○	○	○
Sleep	○	○	○	○	○	○
Pain on intercourse	○	○	○	○	○	○
Pain on urination	○	○	○	○	○	○

Did you experience any adverse reactions from stretching exercises?

Yes

No

What were these adverse reactions?

Did your use of stretching exercises reduce your need to use the normal pharmaceutical therapies you would normally use for your endometriosis symptoms (e.g a reduction in the amount of pain killers you need)?

Yes – significant reduction (50% or more)

Yes – moderate reduction (25-50%)

Yes – minimal reduction (<25%)

No

Other

How much per month, on average, did stretching exercises cost you out of your own money? (This could include a one off costs like a physiotherapy appointment to learn these techniques)

Would you recommend stretching exercises to a friend or family member with endometriosis?

Yes

No

Is there anything else you want to tell us about stretching exercises?

How often did you do exercise or physical activity (such as walking, jogging, swimming or going to the gym) on average?

Daily or multiple times per day

2-6 times a week

Once a week

Less than once a week

On a scale from 0-10 (0 being totally ineffective and 10 being totally effective), how effective was exercise in reducing your endometriosis related pelvic pain?

Totally ineffective									Totally effective	
0	1	2	3	4	5	6	7	8	9	10

Which symptoms of endometriosis, or side effects of medications that you are taking to treat endometriosis, did exercise reduce and how effective was it?

	Significant improvement (> 50% reduction in severity and/or duration)	Moderate improvement (25-50% reduction in severity and/or duration)	Slight improvement (<25% reduction in severity and/or duration)	No change	Felt worse / symptoms flared up	I don' have th sympto
Nausea / Vomiting	○	○	○	○	○	○
Bowel / Stomach problems	○	○	○	○	○	○
Fatigue	○	○	○	○	○	○
Anxiety	○	○	○	○	○	○
Depression / Low mood	○	○	○	○	○	○
Sleep	○	○	○	○	○	○
Pain on intercourse	○	○	○	○	○	○
Pain on urination	○	○	○	○	○	○

Did you experience any adverse reactions from exercise

?

Yes

No

What were these reactions?

Did your use of exercise (such as jogging, swimming or going to the gym) reduce your need to use the normal pharmaceutical therapies you would normally use for your endometriosis symptoms (e.g a reduction in the amount of pain killers you need)?

Yes – significant reduction (50% or more)

Yes – moderate reduction (25-50%)

Yes – minimal reduction (<25%)

No

Other

How much per month, on average, did exercise cost you out of your own money? (This could include the cost of a gym membership for example. If there was no increase put 0)

Would you recommend exercise to a friend or family member with endometriosis?

Yes

No

Is there anything else you want to tell us about exercise (such as what type of

exercise or physical activity you found most helpful)?

How often did you use Heat on average?

Daily or multiple times per day

2-6 times a week

Once a week

Less than once a week

On a scale from 0-10 (0 being totally ineffective and 10 being totally effective), how effective was heat in reducing your endometriosis related pelvic pain?

Totally ineffective									Totally effective	
0	1	2	3	4	5	6	7	8	9	10

Which symptoms of endometriosis, or side effects of medications that you are taking to treat endometriosis, did heat and how effective was it?

	Significant improvement (> 50% reduction in severity and/or duration)	Moderate improvement (25-50% reduction in severity and/or duration)	Slight improvement (<25% reduction in severity and/or duration)	No change	Felt worse / symptoms flared up	I don' have th sympto
Nausea / Vomiting	○	○	○	○	○	○
Bowel / Stomach problems	○	○	○	○	○	○
Fatigue	○	○	○	○	○	○
Anxiety	○	○	○	○	○	○

Depression / Low mood	○	○	○	○	○	○
Sleep	○	○	○	○	○	○
Pain on intercourse	○	○	○	○	○	○
Pain on urination	○	○	○	○	○	○

Did you experience any adverse reactions from using heat?

Yes

No

What were these reactions?

Did your use of heat reduce your need to use the normal pharmaceutical therapies you would normally use for your endometriosis symptoms (e.g a reduction in the amount of pain killers you need)?

Yes – significant reduction (50% or more)

Yes – moderate reduction (25-50%)

Yes – minimal reduction (<25%)

No

Other

How much per month, on average, did using heat cost you out of your own money?

Would you recommend heat to a friend or family member with endometriosis?

Yes

No

Is there anything else you want to tell us about heat, such as what kind of heat you used or which type of heat you found most beneficial ?

How often did you use cold packs on average?

Daily or multiple times per day

2-6 times a week

Once a week

Less than once a week

On a scale from 0-10 (0 being totally ineffective and 10 being totally effective), how effective were cold packs in reducing your endometriosis related pelvic pain?

Totally ineffective										Totally effective
0	1	2	3	4	5	6	7	8	9	10

Which symptoms of endometriosis, or side effects of medications that you are taking to treat endometriosis, did cold packs reduce and how effective was it?

	Significant improvement (> 50% reduction in severity and/or duration)	Moderate improvement (25-50% reduction in severity and/or duration)	Slight improvement (<25% reduction in severity and/or duration)	No change	Felt worse / symptoms flared up	I don' have th sympto

Nausea / Vomiting						
Bowel / Stomach problems						
Fatigue						
Anxiety						
Depression / Low mood						
Sleep						
Pain on intercourse						
Pain on urination						

Did you experience any adverse reactions from cold packs?

Yes

No

What were these reactions?

Did your use of cold packs reduce your need to use the normal pharmaceutical therapies you would normally use for your endometriosis symptoms (e.g a reduction in the amount of pain killers you need)?

Yes – significant reduction (50% or more)

Yes – moderate reduction (25-50%)

Yes – minimal reduction (<25%)

No

Other

How much per month, on average, did cold packs cost you out of your own money?

Would you recommend cold packs to a friend or family member with endometriosis?

Yes

No

Is there anything else you want to tell us about cold packs?

How often did you use acupressure on average?

Daily or multiple times per day

2-6 times a week

Once a week

Less than once a week

On a scale from 0-10 (0 being totally ineffective and 10 being totally effective), how effective was acupressure in reducing your endometriosis related pelvic pain?

Totally ineffective | Totally effective

0	1	2	3	4	5	6	7	8	9	10

Which symptoms of endometriosis, or side effects of medications that you are taking to treat endometriosis, did acupressure reduce and how effective was it?

	Significant improvement (> 50% reduction in severity and/or duration)	Moderate improvement (25-50% reduction in severity and/or duration)	Slight improvement (<25% reduction in severity and/or duration)	No change	Felt worse / symptoms flared up	I don’ have th sympto
Nausea / Vomiting						
Bowel / Stomach problems						
Fatigue						
Anxiety						
Depression / Low mood						
Sleep						
Pain on intercourse						
Pain on urination						

Did you experience any adverse reactions from acupressure?

Yes

No

What were these reactions?

Did your use of acupressure reduce your need to use the normal pharmaceutical therapies you would normally use for your endometriosis symptoms (e.g a reduction in the amount of pain killers you need)?

Yes – significant reduction (50% or more)

Yes – moderate reduction (25-50%)

Yes – minimal reduction (<25%)

No

Other

How much per month, on average, did acupressure cost you out of your own money? (This could include a one off costs like a beginners mediation class. If this is a dietary therapy then the increase over your normal food budget. If there was no increase put 0)

Would you recommend acupressure to a friend or family member with endometriosis?

Yes

No

Is there anything else you want to tell us about acupressure?

How often did you use massages on average?

Daily or multiple times per day

2-6 times a week

Once a week

Less than once a week

On a scale from 0-10 (0 being totally ineffective and 10 being totally effective), how effective were massages in reducing your endometriosis related pelvic pain?

Totally ineffective										Totally effective
0	1	2	3	4	5	6	7	8	9	10

Which symptoms of endometriosis, or side effects of medications that you are taking to treat endometriosis, did massages reduce and how effective was it?

	Significant improvement (> 50% reduction in severity and/or duration)	Moderate improvement (25-50% reduction in severity and/or duration)	Slight improvement (<25% reduction in severity and/or duration)	No change	Felt worse / symptoms flared up	I don' have th sympto
Nausea / Vomiting	◯	◯	◯	◯	◯	◯
Bowel / Stomach problems	◯	◯	◯	◯	◯	◯
Fatigue	◯	◯	◯	◯	◯	◯
Anxiety	◯	◯	◯	◯	◯	◯
Depression / Low mood	◯	◯	◯	◯	◯	◯
Sleep	◯	◯	◯	◯	◯	◯
Pain on intercourse	◯	◯	◯	◯	◯	◯
Pain on urination	◯	◯	◯	◯	◯	◯

Did you experience any adverse reactions from massages?

Yes

No

What were these reactions?

Did your use of massages reduce your need to use the normal pharmaceutical therapies you would normally use for your endometriosis symptoms (e.g a reduction in the amount of pain killers you need)?

Yes – significant reduction (50% or more)

Yes – moderate reduction (25-50%)

Yes – minimal reduction (<25%)

No

Other

How much per month, on average, did massages cost you out of your own money?

Would you recommend massages to a friend or family member with endometriosis?

Yes

No

Is there anything else you want to tell us about massages?

How often did you use hemp/CBD oil on average?

Daily or multiple times per day

2-6 times a week

Once a week

Less than once a week

On a scale from 0-10 (0 being totally ineffective and 10 being totally effective), how effective was hemp/CBD oil in reducing your endometriosis related pelvic pain?

Not effective										Totally effective
0	1	2	3	4	5	6	7	8	9	10

Which symptoms of endometriosis, or side effects of medications that you are taking to treat endometriosis, did hemp/CBD oil reduce and how effective was it?

	Significant improvement (> 50% reduction in severity and/or duration)	Moderate improvement (25-50% reduction in severity and/or duration)	Slight improvement (<25% reduction in severity and/or duration)	No change	Felt worse / symptoms flared up	I don' have th sympto
Nausea / Vomiting	◯	◯	◯	◯	◯	◯
Bowel / Stomach problems	◯	◯	◯	◯	◯	◯
Fatigue	◯	◯	◯	◯	◯	◯
Anxiety	◯	◯	◯	◯	◯	◯
Depression						

/ Low mood	○	○	○	○	○	○
Sleep	○	○	○	○	○	○
Pain on intercourse	○	○	○	○	○	○
Pain on urination	○	○	○	○	○	○

Did you experience any adverse reactions from Hemp/CBD oil?

Yes

No

What were these reactions?

[ ]

Did your use of Hemp/CBD oil reduce your need to use the normal pharmaceutical therapies you would normally use for your endometriosis symptoms (e.g a reduction in the amount of pain killers you need)?

Yes – significant reduction (50% or more)

Yes – moderate reduction (25-50%)

Yes – minimal reduction (<25%)

No

Other

How much per month, on average, did Hemp/CBD oil cost you out of your own money?

[ ]

Would you recommend Hemp/CBD oil to a friend or family member with endometriosis?

Yes

No

Is there anything else you want to tell us about Hemp/CBD oil?

How often did you use cannabis or cannabis products (prescribed or illicit) on average?

Daily or multiple times per day

2-6 times a week

Once a week

Less than once a week

On a scale from 0-10 (0 being totally ineffective and 10 being totally effective), how effective were cannabis or cannabis products in reducing your endometriosis related pelvic pain?

Totally ineffective									Totally effective	
0	1	2	3	4	5	6	7	8	9	10

Which symptoms of endometriosis, or side effects of medications that you are taking to treat endometriosis, did cannabis or cannabis products reduce and how effective was it?

	Significant improvement (> 50%	Moderate improvement (25-50%	Slight improvement (<25%

	reduction in severity and/or duration)	reduction in severity and/or duration)	reduction in severity and/or duration)	No change	Felt worse / symptoms flared up	I don' have th sympto
Nausea / Vomiting	○	○	○	○	○	○
Bowel / Stomach problems	○	○	○	○	○	○
Fatigue	○	○	○	○	○	○
Anxiety	○	○	○	○	○	○
Depression / Low mood	○	○	○	○	○	○
Sleep	○	○	○	○	○	○
Pain on intercourse	○	○	○	○	○	○
Pain on urination	○	○	○	○	○	○

Did you experience any adverse reactions from cannabis or cannabis products?

Yes

No

What were these reactions?

Did your use of cannabis or cannabis products (prescribed or illicit) reduce your need to use the normal pharmaceutical therapies you would normally use for your endometriosis symptoms (e.g a reduction in the amount of pain killers you need)?

Yes – significant reduction (50% or more)

Yes – moderate reduction (25-50%)

Yes – minimal reduction (<25%)

No

Other

How much per month, on average, did cannabis or cannabis products cost you out of your own money?

Would you recommend cannabis or cannabis products to a friend or family member with endometriosis?

Yes

No

What type of cannabis product or form of ingestion did you use (e.g edible, vapouriser) ?

How often did you use alcohol on average?

Daily or multiple times per day

2-6 times a week

Once a week

Less than once a week

On a scale from 0-10 (0 being totally ineffective and 10 being totally effective), how effective was alcohol in reducing your endometriosis related pelvic pain?

Totally ineffective									Totally effective	
0	1	2	3	4	5	6	7	8	9	10

Which symptoms of endometriosis, or side effects of medications that you are taking to treat endometriosis, did alcohol reduce and how effective was it?

	Significant improvement (> 50% reduction in severity and/or duration)	Moderate improvement (25-50% reduction in severity and/or duration)	Slight improvement (<25% reduction in severity and/or duration)	No change	Felt worse / symptoms flared up	I don' have th sympto
Nausea / Vomiting	◯	◯	◯	◯	◯	◯
Bowel / Stomach problems	◯	◯	◯	◯	◯	◯
Fatigue	◯	◯	◯	◯	◯	◯
Anxiety	◯	◯	◯	◯	◯	◯
Depression / Low mood	◯	◯	◯	◯	◯	◯
Sleep	◯	◯	◯	◯	◯	◯
Pain on intercourse	◯	◯	◯	◯	◯	◯
Pain on urination	◯	◯	◯	◯	◯	◯

Did you experience any adverse reactions from alcohol?

Yes

No

What were these reactions?

Did your use of alcohol reduce your need to use the normal pharmaceutical therapies you would normally use for your endometriosis symptoms (e.g a reduction in the amount of pain killers you need)?

Yes – significant reduction (50% or more)

Yes – moderate reduction (25-50%)

Yes – minimal reduction (<25%)

No

Other

How much per month, on average, did alcohol cost you out of your own money? (This could include a one off costs like a beginners mediation class. If this is a dietary therapy then the increase over your normal food budget. If there was no increase put 0)

Would you recommend alcohol to a friend or family member with endometriosis?

Yes

No

Is there anything else you want to tell us about alcohol?

How often did you use herbal anti-anxiety/relaxants (e.g. Kava, Passionflower, Camomile) on average?

Daily or multiple times per day

2-6 times a week

Once a week

Less than once a week

On a scale from 0-10 (0 being totally ineffective and 10 being totally effective), how effective were herbal anti-anxiety/relaxants in reducing your endometriosis related pelvic pain?

Totally ineffective									Totally effective	
0	1	2	3	4	5	6	7	8	9	10

Which symptoms of endometriosis, or side effects of medications that you are taking to treat endometriosis, did herbal anti-anxiety/relaxants reduce and how effective was it?

	Significant improvement (> 50% reduction in severity and/or duration)	Moderate improvement (25-50% reduction in severity and/or duration)	Slight improvement (<25% reduction in severity and/or duration)	No change	Felt worse / symptoms flared up	I don' have th sympto
Nausea / Vomiting	○	○	○	○	○	○
Bowel / Stomach problems	○	○	○	○	○	○
Fatigue	○	○	○	○	○	○
Anxiety	○	○	○	○	○	○
Depression / Low mood	○	○	○	○	○	○
Sleep	○	○	○	○	○	○
Pain on intercourse	○	○	○	○	○	○

Pain on urination	◯	◯	◯	◯	◯	◯

Did you experience any adverse reactions from herbal anti-anxiety/relaxants?

Yes

No

What were these reactions?

Did your use of herbal anti-anxiety/relaxants reduce your need to use the normal pharmaceutical therapies you would normally use for your endometriosis symptoms (e.g a reduction in the amount of pain killers you need)?

Yes – significant reduction (50% or more)

Yes – moderate reduction (25-50%)

Yes – minimal reduction (<25%)

No

Other

How much per month, on average, did herbal anti-anxiety/relaxants cost you out of your own money?

Would you recommend herbal anti-anxiety/relaxants to a friend or family member with endometriosis?

Yes

No

Is there anything else you want to tell us about herbal anti-anxiety/relaxants ?

How often did you use dietary therapies on average?

Daily or multiple times per day

2-6 times a week

Once a week

Less than once a week

On a scale from 0-10 (0 being totally ineffective and 10 being totally effective), how effective were dietary therapies in reducing your endometriosis related pelvic pain?

Totally ineffective ... Totally effective

0 1 2 3 4 5 6 7 8 9 10

Which symptoms of endometriosis, or side effects of medications that you are taking to treat endometriosis, did dietary therapies reduce and how effective was it?

	Significant improvement (> 50% reduction in severity and/or duration)	Moderate improvement (25-50% reduction in severity and/or duration)	Slight improvement (<25% reduction in severity and/or duration)	No change	Felt worse / symptoms flared up	I don' have th sympto
Nausea / Vomiting	○	○	○	○	○	○
Bowel / Stomach	○	○	○	○	○	○

problems						
Fatigue	◯	◯	◯	◯	◯	◯
Anxiety	◯	◯	◯	◯	◯	◯
Depression / Low mood	◯	◯	◯	◯	◯	◯
Sleep	◯	◯	◯	◯	◯	◯
Pain on intercourse	◯	◯	◯	◯	◯	◯
Pain on urination	◯	◯	◯	◯	◯	◯

Did you experience any adverse reactions from dietary therapies ?

Yes

No

What were these reactions?

Did your use of dietary therapies reduce your need to use the normal pharmaceutical therapies you would normally use for your endometriosis symptoms (e.g a reduction in the amount of pain killers you need)?

Yes – significant reduction (50% or more)

Yes – moderate reduction (25-50%)

Yes – minimal reduction (<25%)

No

Other

How much per month, on average, did dietary therapies cost you out of your own money? (This would be any increase over your normal food budget. If there was no increase put 0)

Would you recommend dietary therapies to a friend or family member with endometriosis?

Yes

No

Is there anything else you want to tell us about dietary therapies?

Powered by Qualtrics
