# Supplementary material for: Self-management strategies amongst Australian women with endometriosis: a national online survey
Source: BMC Complement Altern Med. 2019 Jan 15;19:17. doi: 10.1186/s12906-019-2431-x (PMC6332532; doi:10.1186/s12906-019-2431-x)
Supplement: Supplementary file 2 — Table S1. Reduction in medication usage due to the use of self-management. Changes in endometriosis related medication for all self-management modalities. (DOCX 22 kb) [file 12906_2019_2431_MOESM2_ESM.docx]

**Table S1: Reduction in medication usage due to the use of self-management**

| Level of reduction in medication usage | | N (%) |
| --- | --- | --- |
| yoga/Pilates (n = 128) | Significant reduction (50% or more) | 10 (7.8) |
|  | Moderate reduction (25-50%) | 11 (8.6) |
|  | Minimal reduction (<25%) | 30 (23.4) |
|  | No | 75 (58.6) |
|  | Other | 2 (1.56) |
| Tai Chi / Qi Gong (n = 8) | Significant reduction (50% or more) | 0 (0) |
|  | Moderate reduction (25-50%) | 0 (0) |
|  | Minimal reduction (<25%) | 2 (25) |
|  | No | 6 (75) |
|  | Other | 0 (0) |
| Meditation and breathing (n = 166) | Significant reduction (50% or more) | 6 (3.6) |
|  | Moderate reduction (25-50%) | 22 (13.3) |
|  | Minimal reduction (<25%) | 36 (21.7) |
|  | No | 96 (57.8) |
|  | Other | 6 (3.6) |
| Rest (n = 244) | Significant reduction (50% or more) | 10 (4.1) |
|  | Moderate reduction (25-50%) | 32 (13.1) |
|  | Minimal reduction (<25%) | 75 (30.7) |
|  | No | 123 (50.4) |
|  | Other | 4 (1.7) |
| Stretching (n = 141) | Significant reduction (50% or more) | 3 (2.1) |
|  | Moderate reduction (25-50%) | 11 (7.8) |
|  | Minimal reduction (<25%) | 38 (27) |
|  | No | 85 (60.3) |
|  | Other | 4 (2.8) |
| Exercise (n = 156) | Significant reduction (50% or more) | 8 (5.1) |
|  | Moderate reduction (25-50%) | 23 (14.7) |
|  | Minimal reduction (<25%) | 37 (23.7) |
|  | No | 85 (54.5) |
|  | Other | 3 (1.9) |
| Heat (n = 259) | Significant reduction (50% or more) | 25 (9.7) |
|  | Moderate reduction (25-50%) | 60 (23.2) |
|  | Minimal reduction (<25%) | 104 (40.2) |
|  | No | 65 (25.1) |
|  | Other | 5 (1.9) |
| Cold (n = 16) | Significant reduction (50% or more) | 0 (0) |
|  | Moderate reduction (25-50%) | 3 (18.8) |
|  | Minimal reduction (<25%) | 4 (25) |
|  | No | 9 (56.3) |
|  | Other | 0 (0) |
| Acupressure (n = 28) | Significant reduction (50% or more) | 2 (7.1) |
|  | Moderate reduction (25-50%) | 8 (28.6) |
|  | Minimal reduction (<25%) | 8 (28.6) |
|  | No | 9 (32.1) |
|  | Other | 1 (3.6) |
| Massage (n = 118) | Significant reduction (50% or more) | 5 (4.2) |
|  | Moderate reduction (25-50%) | 17 (14.4) |
|  | Minimal reduction (<25%) | 40 (33.9) |
|  | No | 54 (45.8) |
|  | Other | 2 (1.7) |
| Hemp/CBD oil (n = 12) | Significant reduction (50% or more) | 4 (33.3) |
|  | Moderate reduction (25-50%) | 3 (25) |
|  | Minimal reduction (<25%) | 2 (16.7) |
|  | No | 3 (25) |
|  | Other | 0 (0) |
| Cannabis (n = 48) | Significant reduction (50% or more) | 27 (56.3) |
|  | Moderate reduction (25-50%) | 13 (27.1) |
|  | Minimal reduction (<25%) | 6 (12.5) |
|  | No | 2 (4.2) |
|  | Other | 0 (0) |
| Alcohol (n = 53) | Significant reduction (50% or more) | 2 (3.8) |
|  | Moderate reduction (25-50%) | 5 (9.4) |
|  | Minimal reduction (<25%) | 20 (37.7) |
|  | No | 26 (49.1) |
|  | Other | 0 (0) |
| Herbal medicines (n = 62) | Significant reduction (50% or more) | 8 (12.9) |
|  | Moderate reduction (25-50%) | 4 (6.5) |
|  | Minimal reduction (<25%) | 18 (29) |
|  | No | 31 (50) |
|  | Other | 1 (1.6) |
| Diet (n = 155) | Significant reduction (50% or more) | 29 (18.7) |
|  | Moderate reduction (25-50%) | 35 (22.6) |
|  | Minimal reduction (<25%) | 43 (27.7) |
|  | No | 45 (29) |
|  | Other | 3 (1.9) |
